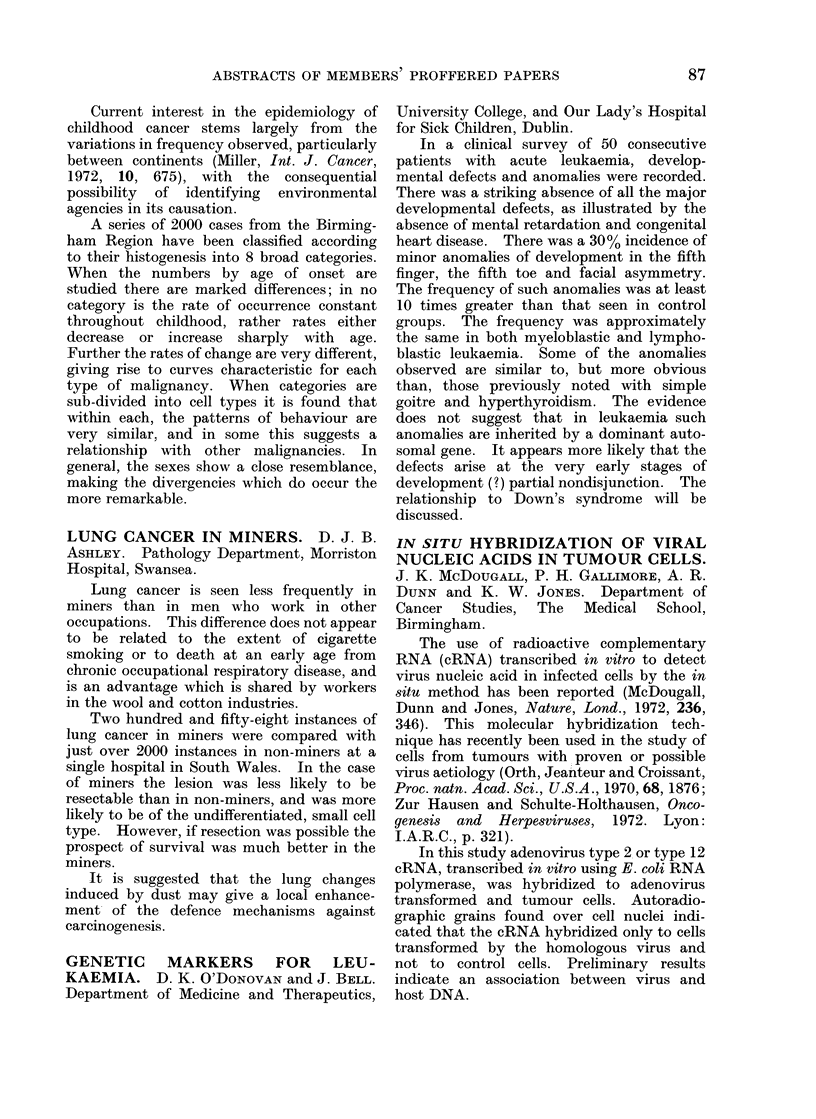# Lung cancer in miners.

**Published:** 1973-07

**Authors:** D. J. Ashley


**LUNG CANCER IN MINERS.** D. J. B. Ashley. Pathology Department, Morriston Hospital, Swansea.

Lung cancer is seen less frequently in miners than in men who work in other occupations. This difference does not appear to be related to the extent of cigarette smoking or to death at an early age from chronic occupational respiratory disease, and is an advantage which is shared by workers in the wool and cotton industries.

Two hundred and fifty-eight instances of lung cancer in miners were compared with just over 2000 instances in non-miners at a single hospital in South Wales. In the case of miners the lesion was less likely to be resectable than in non-miners, and was more likely to be of the undifferentiated, small cell type. However, if resection was possible the prospect of survival was much better in the miners.

It is suggested that the lung changes induced by dust may give a local enhancement of the defence mechanisms against carcinogenesis.